# Gastrointestinal tract involvement in melioidosis

**DOI:** 10.1093/trstmh/trx031

**Published:** 2017-06-28

**Authors:** Prapit Teparrukkul, Worrarat Kongkasame, Songla Chitsaeng, Gumphol Wongsuwan, Vanaporn Wuthiekanun, Sharon J. Peacock, Direk Limmathurotsakul

**Affiliations:** 1 Medical Department, Sunpasitthiprasong Hospital, Ubon Ratchathani, Thailand; 2 Mahidol-Oxford Tropical Medicine Research Unit, Faculty of Tropical Medicine, Mahidol University, Bangkok, Thailand; 3 Department of Medicine, University of Cambridge, Addenbrooke's Hospital, Cambridge, UK; 4 London School of Hygiene and Tropical Medicine, London, UK; 5 Centre for Tropical Medicine and Global Health, Nuffield Department of Medicine, University of Oxford, UK; 6 Department of Tropical Hygiene, Faculty of Tropical Medicine, Mahidol University, Bangkok, Thailand

**Keywords:** *Burkholderia pseudomallei*, Gastrointestinal tract, Melioidosis

## Abstract

**Background:**

Little is known about the involvement of the human gut in carriage and disease associated with *Burkholderia pseudomallei*, the cause of melioidosis.

**Methods:**

A hospital-based study was conducted in Northeast Thailand to culture stools or rectal swabs from patients with melioidosis, stools from controls with non-infectious diseases, and gastric biopsies from patients undergoing routine endoscopic investigation.

**Results and Conclusion:**

*B. pseudomallei* was isolated from 9/83 (11%) stools and 9/58 (16%) rectal swabs from 141 patients with melioidosis. All stools from 244 control patients and 799 gastric biopsies from 395 patients with no evidence of melioidosis were culture negative for *B. pseudomallei*. It is not uncommon for melioidosis patients to shed *B. pseudomallei* in stool. Colonization of the gut of individuals without signs and symptoms of melioidosis may be rare.

## Introduction

Melioidosis is an often fatal infectious disease caused by the Gram-negative bacillus *Burkholderia pseudomallei*, which is commonly found in soil and water in tropical countries.^1^ The disease is highly endemic in Southeast Asia and Northern Australia, and is increasingly reported from many other tropical regions, including South Asia, Africa, and Central and South America.^1^ A recent modeling study estimated that there are about 165 000 human melioidosis cases per year worldwide, of whom 89 000 (54%) die.^1^ Skin inoculation is considered to be the main route of infection, although there is increasing evidence to suggest that ingestion is an important route of human infection.^2,3^

In experimental mouse models, *B. pseudomallei* ingestion produces an effective infection, is associated with colonization of the stomach, results in acute or chronic disease depending on inoculating dose, and may lead to gut carriage and shedding in the stool for weeks after inoculation.^4,5^*B. pseudomallei* can be isolated from rectal swabs during acute human infection,^6,7^ which are routinely collected from patients with suspected melioidosis in Australia.^8^ However, it is not known whether humans with melioidosis develop gut colonization and shed *B. pseudomallei* in the stool. Furthermore, *B. pseudomallei* is likely to be frequently consumed in water and food in settings where the organism is present in the environment,^2,3^ and could colonize the gastrointestinal tract without clinical features or prior to the clinical presentation of melioidosis. A recent study conducted in Malaysia reported the isolation of *B. pseudomallei* from gastric biopsy samples in 15 of 215 (7%) patients who had gastric biopsy samples tested for *Helicobacter pylori.*^9^

We previously conducted a prospective hospital-based 1:2 matched case-control study to investigate the activities of daily living associated with acquisition of melioidosis in Northeast Thailand.^2^ Here, we reported the prevalence of *B. pseudomallei* in feces and rectal swabs taken from patients with melioidosis and in feces of control patients. In addition, we investigated whether *B. pseudomallei* colonizes the stomach in humans without clinical signs and symptoms of melioidosis by culturing gastric biopsies taken from patients in Northeast Thailand.

## Materials and methods

A total of 83 stools and 58 rectal swabs were obtained from 141 culture-confirmed melioidosis cases presenting to Sunpasitthiprasong Hospital, Ubon Ratchathani, Northeast Thailand, between July 2010 and December 2011.^2^ A single sample was taken from each case, with rectal swabs collected in cases who were critically ill. In parallel, 244 stool samples were collected from 244 patients who were admitted to the study hospital with non-infectious conditions during the same period (controls).^2^ We also collected 799 gastric biopsy samples from 395 patients who underwent endoscopy and gastric biopsy at the study hospital between June 2014 and December 2015. The majority (n=390, 99%) had two gastric samples, the remaining cases having either one sample (1 patient), three samples (4 patients) or six samples (1 patient).

Rectal swabs were inoculated onto Ashdown selective agar and then placed into CVC50 broth (trypticase soy broth with crystal violet at 5 mg/liter plus colistin at 50 mg/liter).^8^ For each stool sample, an applicator swab was used to collect a small amount of stool from the container and directly inoculated onto Ashdown agar, and around 5 g of stool was added to CVC50 broth. Gastric biopsy samples were inoculated into CVC50 broth. All broths were incubated at 37°C in air for 48 hours, after which 10 μl was sub-cultured onto Ashdown agar. All agar plates were incubated at 37**°**C in air and inspected visually for 4 days. Presumptive *B. pseudomallei* colonies were identified using established methodologies.^8^

## Results and Discussion

*B. pseudomallei* was isolated from 9/83 (11%) stools and 9/58 (16%) rectal swabs from 141 patients with melioidosis, confirming that it is not uncommon for these patients to shed *B. pseudomallei* in stool. The median age of patients was 53 years (range 18–78 years), 91 (65%) were men, 123 (87%) were considered acute (defined as symptoms present for less than 2 months), and 28 (20%) died within 28 days of the admission date. *B. pseudomallei* was also isolated from blood (n=67; 48%), respiratory specimens (61; 43%), urine (n=25; 18%), pus (n=47; 33%), throat swab (n=55; 39%), swabs from skin lesioins (n=10; 7%) and synovial fluid (n=7; 5%). This observation highlights an important possible reservoir for the introduction of *B. pseudomallei* into non-endemic areas, and may explain the mechanism by which this bacterium became globally disseminated between 1650 and 1850.^10^ Duration of carriage is unknown, and it is not clear whether *B. pseudomallei* in stool and the rectal mucosa originated from primary gastrointestinal infection following ingestion or from secondary dissemination following inoculation or inhalation. It is also possible that *B. pseudomallei* simply passed through the gastrointestinal tract after patients swallowed sputum or drank contaminated water.

All stools from 244 controls with non-infectious diseases and 799 gastric biopsies from 395 patients with no clinical evidence of melioidosis were culture negative for *B. pseudomallei*. This fails to replicate previous findings of positive gastric biopsies in people residing in a region where melioidosis is endemic,^9^ and indicates that *B. pseudomallei* colonization of the gut of individuals without signs and symptoms of melioidosis is rare in Northeast Thailand despite frequent ingestion.^2,3^ Further studies are needed to evaluate the presence of *B. pseudomallei* in drinking water, food and the human stomach in Kuala Lumpur and elsewhere in Malaysia.
